# Mechanical Characteristics of Multi-Level 3D-Printed Silicone Foams

**DOI:** 10.3390/ma17164097

**Published:** 2024-08-19

**Authors:** Zhirong Yang, Jinpeng Wen, Guoqi Zhang, Changyu Tang, Qingtian Deng, Jixin Ling, Haitao Hu

**Affiliations:** 1 Institute of Systems Engineering, China Academy of Engineering Physics, Mianyang 621999, China; zhirong.yang@caep.cn (Z.Y.); 401wenjp@caep.cn (J.W.); zhangflag04@163.com (G.Z.); lingjixin@foxmail.com (J.L.); 2 Chengdu Development Center of Science and Technology, China Academy of Engineering Physics, Chengdu 610200, China; tangcy0001@yinhe596.cn; 3 School of Science, Chang’an University, Xi’an 710064, China; dengqt@chd.edu.cn

**Keywords:** silicone foams, 3D printed, mechanical properties, microstructure

## Abstract

Three-dimensional-printed silicone rubber foams, with their designable and highly ordered pore structures, have shown exceptional potential for engineering applications, particularly in areas requiring energy absorption and cushioning. However, optimizing the mechanical properties of these foams through structural design remains a significant challenge. This study addresses this challenge by formulating the research question: How do different 3D-printed topologies and printing parameters affect the mechanical properties of silicone rubber foams, and how can we design a novel topological structure? To answer this, we explored the mechanical behavior of two common structures–simple cubic (SC) and face-centered tetragonal (FCT)–by varying printing parameters such as filament spacing, filament diameter, and layer height. Furthermore, we proposed a novel two-level 3D-printed structure, combining SC and FCT configurations to enhance performance. The results demonstrated that the two-level SC-SC structure exhibited a specific energy absorption of 8.2 to 21.0 times greater than the SC structure and 2.3 to 7.2 times greater than the FCT structure. In conclusion, this study provides new insights into the design of 3D-printed silicone rubber foams, offering a promising approach to developing advanced cushioning materials with superior energy absorption capabilities.

## 1. Introduction

Silicone rubber foam materials (referred to as silicone foams) have been used as cushion materials for the aforementioned mechanical buffering and regulation due to their excellent physical, chemical, and compressible properties [1,2,3,4]. Therefore, they have been widely applied in fields, such as aerospace, weapons and equipment, transportation, energy and chemical, and biomedical fields, involving vibration damping, protection, noise reduction, heat insulation, fire resistance, separation, filtration, and tissue repair, among other uses [5,6,7,8,9]. They occupy an important position in various engineering applications.

The mechanical properties of silicone foams are not only related to the mechanical properties of the base material but also to the pore structure [10,11,12]. The preparation method mainly affects the micropore structural characteristics of the foam material and consequently affects the macroscopic mechanical properties. Traditional preparation methods can be divided into physical mechanisms [13,14] and chemical mechanisms [15,16] according to the pore formation method. However, the pore structure of traditional silicone rubber foams is uncontrollable, leading to high non-uniformity in pore size, shape, and interconnectivity, resulting in randomness in macroscopic compressive mechanical properties, which greatly affects the design and regulation of their mechanical behavior.

With the rapid development of 3D printing technology, ordered open-pore silicone foam materials with designable and highly ordered pore structures have been successfully printed using direct-write additive manufacturing equipment [17,18]. This method allows for a more precise control of the pore structure, meeting the current engineering demand for customizable mechanical properties of silicone foams. Additionally, the uniform structure leads to a more uniform compressive stress than traditional random structures, resulting in outstanding mechanical properties [19,20].

In 2014, Duoss et al. [17] from the Lawrence Livermore National Laboratory chose Dow Corning’s SE1700 silicone rubber material suitable for direct-write 3D printing, and based on a high-precision motion printing platform, they fabricated two classic silicone foams: simple cubic (SC) and face-centered tetragonal (FCT) structure. This method involves extruding rubber ink into filaments and stacking them layer-by-layer to form a mesh structure, followed by thermal curing and crosslinking. The formation process and rubber vulcanization process are separated, allowing for a more precise control of the pore structure (including the size and shape of pores and pore walls). Maiti et al. conducted stress relaxation experiments on FCT-type 3D-printed and randomly foamed materials [21]. It was found that FCT-type silica aerogels have better stress relaxation performance. Based on the parameters of the ratio of filament spacing to spacing and the compression modulus of the materials, a normalized curve fitting was performed by Weisgraber et al. to establish a constitutive model for 3D-printed foams [22]. Zhu et al. printed silicone rubber filaments of different diameters by setting and adjusting the printing conditions and established a theoretical prediction model for the printed filaments based on comprehensive extrusion theory [20]. This enables a more precise preparation of the desired direct-write 3D-printed silicone foam structures, shortening the design and adjustment cycle of silicone foam structures.

For applications as a superior cushioning or damping material, silicone foams must be engineered with an extended stress plateau region to effectively restrict excessive fluctuations in stress amplitude [23]. An expanded plateau region enables elastomeric foams to exhibit enhanced energy absorption capabilities, minimizing stress spike magnitudes when subjected to vibratory conditions.

In this study, a direct-write 3D printing platform and modified silicone rubber ink with good rheological properties were used to print silicone foams with SC and FCT structures. Compression tests were conducted to analyze the effect of the printing parameters on compressive mechanical properties. Based on the pore characteristics of the silicone foam materials, multi-level 3D-printed configurations were proposed. Quasi-static uniaxial compression analyses were performed on the optimized configurations. The specific energy absorption capability of multi-level 3D printing was compared.

## 2. Experimental Details

A two-component silicone rubber ink was used: Component A was the silicone rubber base, and component B was the diluent. The ink was prepared by mixing components A and B in a ratio of 10:1. During the preparation of the printing paste, 3-butyn-1-ol ($\mathrm{HC}\equiv {\mathrm{CCH}}_{2}{\mathrm{CH}}_{2}\mathrm{OH}$) was added as an inhibitor to prevent premature curing of the silicone rubber. Component A, Component B, and the inhibitor were mixed in a ratio of 100:10:1 and centrifuged at 2000 rpm for 30 min. The mixture was then poured into the printing paste tube and centrifuged again at 8000 rpm for 15 min to remove air bubbles. Finally, the syringe tip was sealed to prevent the paste from spilling, and the paste was stored in a refrigerator for use.

**Figure 1:**
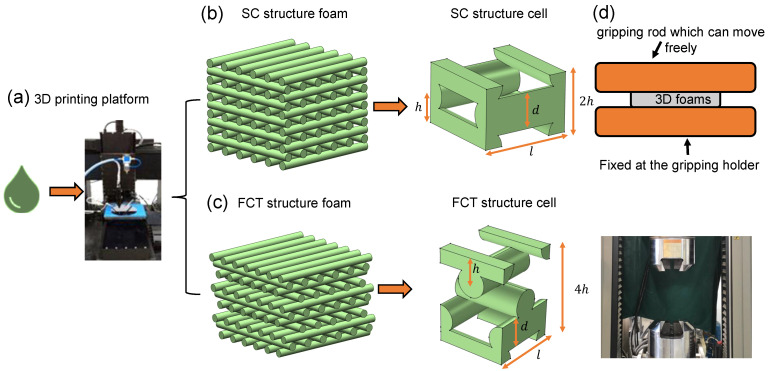
Preparation and testing process for 3D-printed silicone rubber foam. (**a**) The 3D printing platform. (**b**) Simple cubic structure (SC structure). (**c**) Face-centered tetragonal (FCT structure). (**d**) Illustration of quasi-static compression experiment.

A linear direct-write 3D printing motion platform suitable for silicone rubber printing at room temperature was set up, as shown in Figure 1a. The platform mainly consists of a computer-aided design system, a three-dimensional motion system, and a material delivery system. The printing paste is delivered to a screw valve via air pressure, where the rotating screw shears and extrudes the silicone rubber through the nozzle. The nozzle, attached to the motion device, moves along a programmed path in the plane. After each layer is printed, the *z*-axis raises the platform to the specified height, and the process is repeated until the entire structure is formed. In the environment at 20 °C, the extrusion pressure from the gas cylinder is set to 33 psi, and a 2 mm inner diameter nozzle is used to print samples with varying structures, filament spacing, filament diameters, and layer counts. After printing, all samples are placed in an oven and cured with forced air at 150 °C for 40 min. The investigation focused on SC and FCT structures, as illustrated in Figure 1b,c. The mechanical properties of these silicone foam structures were anticipated to be governed by various printing parameters, namely, the spacing between adjacent filaments in the same plane (*l*), the filament diameter (*d*), the layer-to-layer distance or height (*h*), and the total number of printed layers (*n*). A series of samples were fabricated, with each specimen designated, for example, by FCT-8-0.6-7, where FCT specifies the topology structure, 8 represents the number of layers, 0.6 corresponds to the filament spacing, and 7 denotes the printing velocity. The velocity of 6 mm/s and 7 mm/s corresponds to filament diameters of (0.242 ± 0.015) mm and (0.232 ± 0.014) mm, respectively.

The optical microscope was used to observe the microstructure of the printed foam. The mechanical testing and analysis of the silicone rubber foam in this study were conducted using a CMT5305 electronic universal testing machine. For compression testing, the samples were loaded at a rate of 0.5 mm/min until reaching 70% strain, enabling the acquisition of stress–strain curves throughout the loading process shown in Figure 1d. To gain insights into the local stress distributions within the compressed 3D-printed foams, the finite element method (FEM) was performed. The silicone foam was assumed to be unconstrained, with free boundary conditions applied. Rigid plates were affixed to the top and bottom surfaces, facilitating compression through linear displacement of the upper plate while the lower plate remained fixed. The material properties were derived by fitting the experimental data from the 3D silicone matrix to the Mooney–Rivlin constitutive model.

## 3. Results and Discussion

### 3.1. The Effect of Layers and Topological Structure on the Mechanical Properties

**Figure 2:**
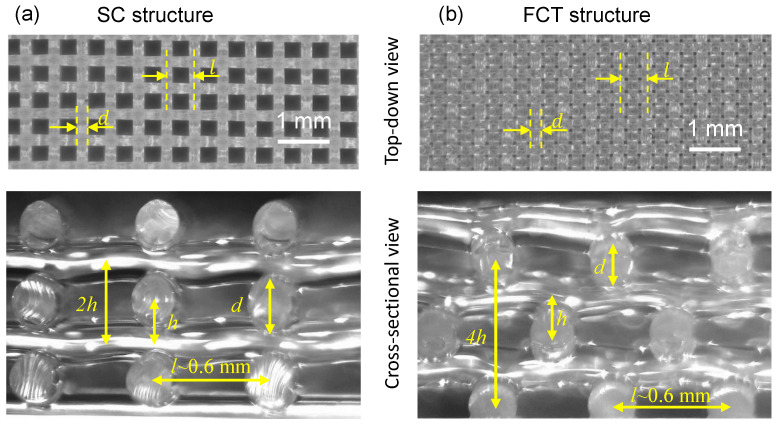
The microstructure of 3D-printed silicone rubber foam. (**a**) Images of direct-write 3D-printed SC silicone foam. (**b**) Images of direct-write 3D-printed FCT silicone foam.

As shown in Figure 2, 3D-printed silicone rubber foams of SC and FCT types are depicted. It can be observed that the printed filament diameters *d* and spacing *l* between filaments are distributed uniformly, indicating stable control over the printing process. From the cross-sectional views, the actual structures of SC and FCT are almost identical to the models described in Figure 1. The main differences lie in the presence of localized deformations at the filament junctions and slight bending of the filaments due to gravity in the actual printed structures. However, these deformations are minimal and, therefore, do not affect the structural mechanical analysis. Therefore, the subsequent analyses do not consider mechanical performance differences caused by printing accuracy.

**Figure 3:**
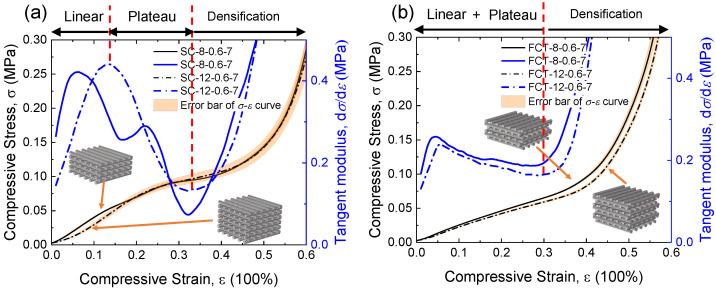
Stress–strain curves and tangent modulus curves with different layers *n* of (**a**) SC structure and (**b**) FCT structure.

The stress–strain curves of 3D-printed silicon foams with different structures and layers are shown in Figure 3. The tangent modulus (blue line) was calculated by E=dσ/dε, where σ is the compressive stress and ε is the compressive strain. It can be seen that as the strain increases, the tangent modulus *E* initially increases, then decreases, and finally continues to rise. Hence, the stress–strain curves of SC and FCT structures can be divided into linear, plateau, and densification regions. The curve of the SC structure undergoes a more pronounced transition at the beginning and end of the plateau region. In contrast, the FCT-type structure does not exhibit an inflection point in the plateau region, and the curvature of the curve in the plateau region is roughly equivalent to that in the linear region. Before densification, the tangent modulus of the SC type decreases from 0.44 MPa to 0.07 MPa, while that of the FCT type decreases from 0.26 MPa to 0.16 MPa. This indicates that, due to different deformation modes, the FCT type appears softer compared to the SC type before densification. However, after the densification point, for example, at a 40% strain, the tangent modulus of the FCT type is 0.50 MPa, whereas the SC type is 0.24 MPa. Therefore, the FCT type appears stiffer than the SC type after densification.

During the initial compression of the predominantly axially compressed SC-type structure, the load is transmitted vertically between structural units forming what is referred to as “stress columns” [17], which are stiff but unstable. Instability buckling occurs as it enters the plateau region, as specifically shown in Figure 3a by the first “inflection point” in strain. The FCT structure primarily undergoes bending deformation in its initial compressed state. The load is redistributed as the compression increases, pressing suspended filaments of each layer into the pores of the lower layers, redistributing the load at the contact area of each layer, and maintaining consistency in the deformation process in both the linear and plateau regions. At a certain point, both structures begin to enter the densification stage, where interlayer contact begins to alter the structural load-bearing mode, resulting in inflection points in the stress–strain curve and a sharp increase in stress.

The influence of the number of layers *n* on the mechanical properties (dot black line) is shown in Figure 3. For the FCT structure, increasing the number of layers has no significant effect on stress transmission, except for localized effects on friction between the foam ends and the plate. However, for the SC structure, increasing the number of layers will elongate the “stress columns”, thereby increasing the flexibility of the members, potentially leading to an early entry into the stress plateau region. Nevertheless, experimental results indicate that within the range of 8 to 12 layers, the number of layers has no significant effect on the “inflection point” between the linear stage and the plateau stage.

### 3.2. The Effect of Filament Spacing and Diameter on the Mechanical Properties

**Figure 4:**
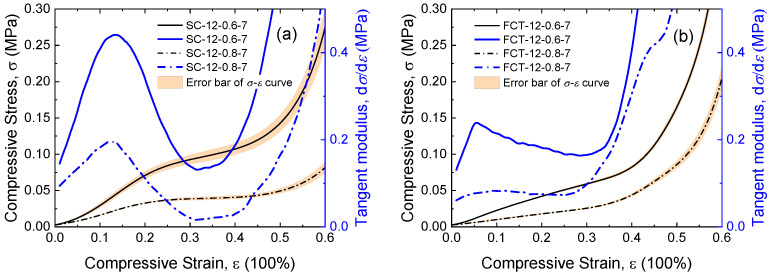
Stress–strain curves and tangent modulus curves with different filament spacings *l* of the (**a**) SC structure and (**b**) FCT structure.

The influence of filament spacing *l* on silicone foam is shown in Figure 4. For both the SC and FCT structures, the initial modulus in the linear region decreases with an increase in filament spacing *l*. Regarding the influence of spacing on the compressive properties of the FCT type, at 40% strain, the foam with a spacing of 0.6 mm has a stress of 0.08 MPa and a tangent modulus of 0.30 MPa, while the foam with a spacing of 0.8 mm has a stress of 0.04 MPa and a tangent modulus of 0.42 MPa. In the case of the SC type, at 40% strain, the foam with a spacing of 0.6 mm has a stress of 0.10 MPa and a tangent modulus of 0.11 MPa, while the foam with a spacing of 0.8 mm has a stress of 0.04 MPa and a tangent modulus of 0.02 MPa. Specifically, within the SC structure, the number of stress columns borne within the foam decreases with an increase in filament spacing, while within the FCT structure, the bending stiffness of the filaments within the unit decreases with an increase in filament spacing.

**Figure 5:**
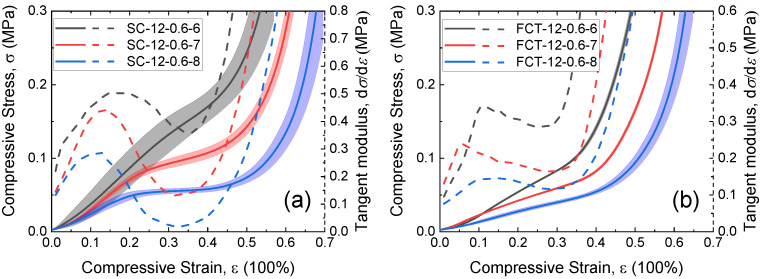
Stress–strain curves and tangent modulus curves with different filament diameters *d* of the (**a**) SC structure and (**b**) FCT structure.

Figure 5 presents a comparison of the compressive stress–strain curves of the SC and FCT structures with different filament diameters *d*. It can be observed that the initial compressive modulus *E* increases with the increase in filament diameter *d* for both structures. Regarding the influence of spacing on the compressive properties of the SC type, at 40% strain, as the filament diameter increases, the stress increases from 0.06 MPa to 0.17 MPa. In the FCT type, the stress increases from 0.05 MPa to 0.14 MPa. Specifically, for the SC structure, increasing the filament diameter *d* widens the thickness of the cell wall, thereby enhancing the load-bearing capacity. Meanwhile, as the filament diameter increases, the volume of the stress column region in the structure expands, which intuitively enhances the buckling strain, given the buckling instability mechanism. For the FCT structure, increasing the filament diameter *d* increases the bending stiffness of the filament within the cell. The effect of filament diameter on the inflection point strain of the FCT structures is relatively small. The modulus and densification point of these structured porous materials can be simplified into the Gent-Thomas model [24,25] and the Gibson–Ashby model [26], where the compressive properties are related to the structure directly. Weisgraber et al. explored the mechanical properties of different SC-type and FCT-type structures based on this concept [22].

### 3.3. FEM Modeling of 3D-Printed Silicon Foam

**Figure 6:**
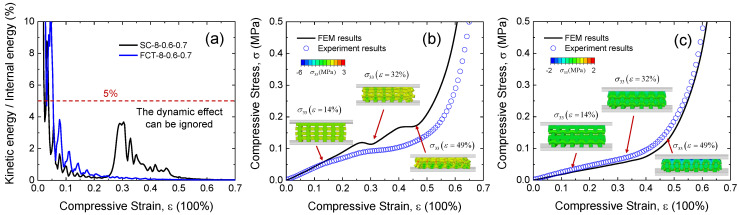
Comparison and analysis of finite element and experimental results. (**a**) Comparison between kinetic energy and internal energy in explicit method. (**b**) Comparison of the stress–strain curve of SC-8-0.6-7 between the experiment and FEM. (**c**) Comparison of the stress–strain curve of FCT-8-0.6-7 between the experiment and FEM.

The finite element method was used to reveal the microscopic deformation mechanism behind the mechanical properties. Figure 6 shows the finite element models of two types of silicon foam structures (SC-8-0.6-7 and FCT-8-0.6-7). Furthermore, in the experiment, the stiffness of the pressure plate is much greater than that of the silicon foam material. Therefore, in the finite element model, the upper and lower pressure plates are set as rigid bodies. The upper-pressure plate adds a displacement load in the compression direction, namely, the third direction, and the lower pressure plate applies fixed constraints. The remaining directions of the structure are set as free boundary conditions. During the compression process, the porous model includes self-contact and contact between the structure and the pressure plate. Therefore, an explicit method and general contact properties are adopted, defining the frictional properties in the form of penalty functions, with the normal direction defined as hard contact and the coefficient of friction set to 0.4 in the tangential direction.

The mechanical properties of the silicone rubber materials are described using the Mooney–Rivlin hyperelastic constitutive model. Its fully incompressible form is shown by

(1) $$\begin{array}{c} W=C_{10}\left(I_{1}-3\right)+C_{01}\left(I_{2}-3\right) \end{array},$$

where *W* is the strain energy density function of the material, $C_{10}$ and $C_{01}$ are fitting parameters of the material, and $I_{1}$ and $I_{2}$ are the first and second principal invariants of the strain tensor driven by

(2) $$\begin{array}{c} I_{1}=\lambda^{2}+\frac{2}{\lambda } \end{array},$$

(3) $$\begin{array}{c} I_{2}=2\lambda +\frac{2}{\lambda^{2}} \end{array},$$

where λ=1+ε. Hence, the $C_{10}$ and $C_{01}$ parameters of the Mooney-Rivlin hyperelastic constitutive model can be obtained by fitting the uniaxial stress–strain curve

(4) $$\begin{array}{c} \sigma =2C_{10}\left(\lambda -\frac{1}{\lambda^{2}}\right)+2C_{01}\left(1-\frac{1}{\lambda^{3}}\right) \end{array}$$

of solid silicone rubber. The silicone rubber ink was prepared in a solid cylinder with a height of 12.5 mm and a diameter of 29 mm. The parameters for fitting Equation (4) were obtained as follows: $C_{10}$ = 0.5112 MPa and $C_{10}$ = 0.01086 MPa.

To address the significant time-consuming issues in an explicit analysis, the structural mass scaling was used. Figure 6a shows the processing results of the energy response during the compression process of the SC and FCT structures, with a horizontal dashed line (5%) added for a comparative analysis. It is found that within the initial compression interval, the ratio of kinetic energy to internal energy rises rapidly, but after that, the ratio curves of both structures quickly drop below 0.05 and approach 0. Hence, the selection of the scaling factor and loading duration in this study is considered reasonable.

Figure 6b,c provide stress–strain curves and stress contour plots ($\sigma_{33}$) of the SC and FCT structures in the compression direction. When compressive strain ε = 14%, the stress in SC structure is transmitted along the layer overlap region, forming a vertical stress column, which is indicative of axial compression deformation as the primary load-bearing mechanism. Due to its interleaved arrangement, the stress in the FCT structure disperses at the contact areas of each layer, which is primarily dominated by bending deformation. When compressive strain ε = 32%, evident instability is observed in the stress columns of the SC structure from the contour plot, with the buckling behavior pulling the middle-layer structure in the transverse direction. The FCT structure continues its bending deformation mode at this stage, exhibiting a wavy structure along the transverse direction. When compressive strain ε = 49%, the stress column structure in the SC structure disappears, and the interlayer contact becomes intimate, leading to a change in the load-bearing mode. Under the same strain, the FCT structure transitions from a bending deformation mode to a densification phase through a dense interlayer contact.

The FEM results of both the SC and FCT structures align well with experimental data, demonstrating consistency between FEM-revealed deformation mechanisms and experimental observations. This confirms the correctness of the finite element analysis. Building upon this foundation, the study continues to explore a novel topology structure.

### 3.4. A Multi-Level 3D-Printed Silicone Foam

It has been observed that a 3D-printed silicon foam enters a densification point at around 50% strain, after which the stress sharply increases. This is primarily due to contact between the filaments, changing the structural deformation to filament deformation itself. Therefore, if the filaments can be designed as a multi-layer printed structure, after reaching the densification point, the deformation of the filaments itself will induce the deformation of the internal multi-layer structure, thereby forming a secondary buckling stage. This significantly broadens the stress plateau region of the 3D foam. This entity is constructed using multi-level stacked basic cells, resulting in a fractal-like porous structure.

**Figure 7:**
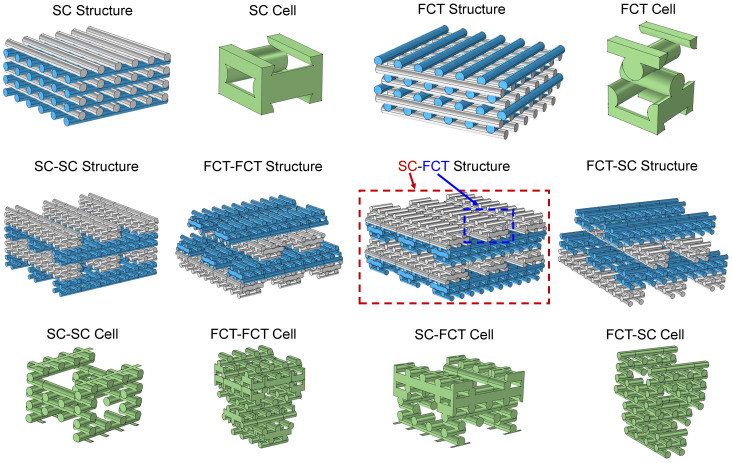
Comparison of different 3D-printed structures and cells, which are SC, FCT, SC-SC, FCT-FCT, SC-FCT, and FCT-SC cell.

The mechanical performances of six different 3D-printed structures are compared, as shown in Figure 7, which are SC, FCT, SC-SC, FCT-FCT, SC-FCT, and FCT-SC. The SC and FCT structures are consistent with those described earlier. SC-FCT means that from a macroscopic perspective, the structure is of the SC structure, while the filaments are of the FCT structure. To reduce the computational cost, this study comparatively analyzes the mechanical performance differences between the SC-SC, FCT-FCT, SC-FCT, and FCT-SC cell structures and the single SC and FCT cell shown in Figure 7. We can infer the mechanical properties of the corresponding macroscopic 3D-printed structures from the mechanical responses of cells, where the SC and FCT cells correspond to the macroscopic SC and FCT structures. Other cells correspond to macroscopic multi-level 3D-printed structures. In the FEM process, the kinetic energy of the system is less than 5% of the internal energy, so it can be regarded as an approximately quasi-static compression process. Controlling the topology of 3D-printed silicone rubber is an important issue. Researchers have explored this by selecting different unit cells, such as the pyramid structure [27] and the Kagome structure [28]; different filament shapes (e.g., circular [29] and square [30]); or by adjusting the unit cell parameters at different positions within the printed structure, resulting in a non-uniform distribution of the printed silicone rubber [19]. The approach of this study is to make the basic units that constitute 3D-printed silicone rubber (such as the aforementioned filaments) themselves into structures formed by stacking basic units. As a result, it possesses a multilevel printed structure, which can create staged cell collapse, offering a new design concept for silicone rubber printing structures.

**Figure 8:**
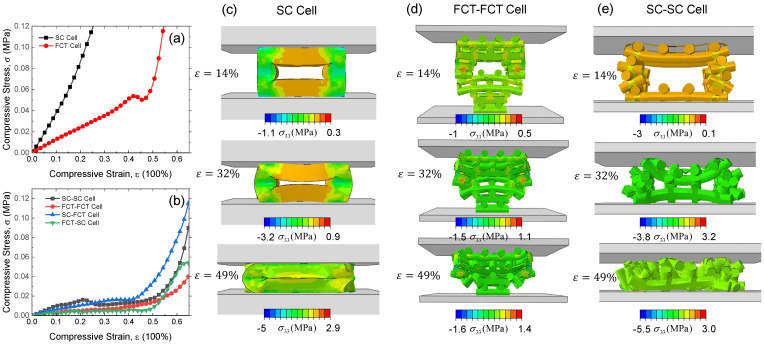
Compression behavior of silicone rubber with different hierarchical structures. (**a**) Mechanical properties of SC and FCT cells. (**b**) Mechanical properties of SC-SC, FCT-FCT, SC-FCT, FCT-SC cells. (**c**) Deformation and stress $\sigma_{33}$ contour of SC cell. (**d**) Deformation and stress $\sigma_{33}$ contour of FCT-FCT cell. (**e**) Deformation and stress $\sigma_{33}$ contour of SC-SC cell.

The mechanical response of different cells is shown in Figure 8. The SC cell has a higher compression modulus due to the axial compression of the stress columns, and the deformation mode of the FCT cell is the bending of the filaments, resulting in a lower compression modulus. It was found that the compression curve of the silicon rubber foam with a multi-level structure is flatter than those of the FCT and SC cells, exhibiting a more pronounced stress plateau region. Under a specified stress, it undergoes larger deformation and exhibits superior energy absorption capability.

**Figure 9:**
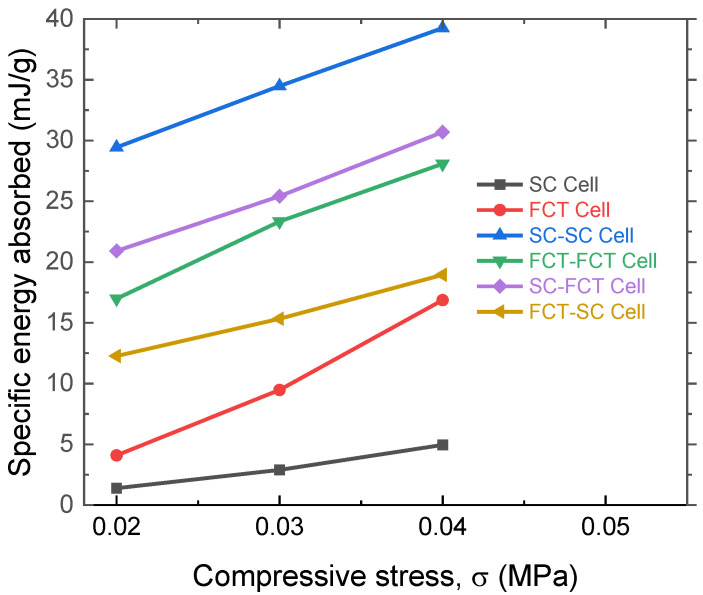
Comparison of specific energy absorption capabilities of different 3D-printed cells, which are SC, FCT, SC-SC, FCT-FCT, SC-FCT, and FCT-SC structures.

The specific energy absorption (SEA) capabilities of the six structures are compared in Figure 9. Under stresses of 0.2, 0.3, and 0.4 MPa, the specific energy absorption of the SC-SC structure is 29.4, 34.5, and 39.3 mJ/g, respectively. In comparison, the SEA for the SC structure is 1.4, 2.9, and 4.9 mJ/g, while for the FCT structure, it is 4.1, 9.5, and 16.9 mJ/g. The SC cell shows the worst energy absorption performance, where the high modulus of the SC structure leads to poor deformability under load as shown in Figure 8c, resulting in a poor energy absorption ability. In contrast, the SC-SC structure exhibits the best energy absorption capability. This is because it combines the advantage of the good load-bearing capacity of the SC structure with the rich multi-level porous structure, enabling continuous energy absorption through pore deformation. The FCT-FCT structure has a relatively lower energy absorption level than the SC-SC structure, where the deformation in both levels is primarily bending, and although it has a longer stress plateau, the plateau stress level is relatively low, resulting in a non-optimal energy absorption level. In summary, the energy absorption capability of 3D-printed silicon rubber foams is influenced by both the stress and width of the plateau region. While maintaining a certain stress plateau height, achieving a wider stress plateau is the key to realizing high specific energy absorption. How to control the printing parameters of the multi-level structures to obtain optimal specific energy absorption performance is currently under investigation.

## 4. Conclusions

This study utilized direct ink-writing 3D printing technology to design and manufacture ordered silicone foams with two distinct woodpile structures, namely, the stretching-dominated SC structure and the bending-dominated FCT structure. The findings reveal that the SC structure primarily bears compressive loads through axial compression of its columnar elements, while the deformation mode of the FCT structure is dominated by the bending of its slender members. The study found that a smaller filament spacing and larger filament diameters result in stiffer silicone foams. To broaden the stress plateau region of silicone rubber foams, this work proposed a hierarchical 3D-printed silicone rubber structure. Compared to the FCT and SC structures, this hierarchical structure enables continued pore compression deformation of the silicone rubber even after reaching the densification point, further widening the stress plateau region. Under a specific load, its specific energy absorption capability far exceeds that of the SC and FCT structures. The specific energy absorption of the two-stage SC-SC structure proposed in this study is 8.2 to 21.0 times that of the SC structure and 2.3 to 7.2 times that of the FCT structure. However, optimizing the parameters of the multi-level structure to achieve the desired mechanical properties is not included within the scope of this study. Meanwhile, the mechanical performance of the multi-level structure is analyzed through a finite element model, and a comparative analysis between the conclusions drawn from the finite element analysis and experimental results is still ongoing. The main innovation of this study lies in utilizing the multi-level printed structure to achieve pore collapse at different stages, thereby creating favorable conditions for enhancing the cushioning performance of silicone rubber (by expanding the width of the stress plateau region). The multi-level topological structure proposed in this study is particularly significant for cushioning design in precision devices.

## Data Availability

The original contributions presented in the study are included in the article, and further inquiries can be directed to the corresponding author.
